# Comment on “On Soft *β*-Open Sets and Soft **β**-Continuous Functions”

**DOI:** 10.1155/2015/515939

**Published:** 2015-05-28

**Authors:** A. K. Mousa, A. Ghareeb

**Affiliations:** ^1^Department of Mathematics, Faculty of Science, Al-Azhar University, Asyut 71524, Egypt; ^2^Department of Mathematics, Faculty of Science, South Valley University, Qena 83523, Egypt

Akdag and Ozkan pointed out in [1, Example 1] that the collection *τ* of soft sets is a soft topology over the universe *X* = {*x*_1_, *x*_2_, *x*_3_} where *E* = {*e*_1_, *e*_2_, *e*_3_} is the set of parameters. This conclusion is not correct since $(F_{2},E)\tilde{\cap }(F_{7},E)\notin \tau$, $(F_{3},E)\tilde{\cup }(F_{8},E)\notin \tau$, $(F_{1},E)\tilde{\cup }(F_{13},E)\notin \tau$, $(F_{1},E)\tilde{\cap }(F_{13},E)\notin \tau$, $(F_{2},E)\tilde{\cap }(F_{13},E)\tilde{\cap }(F_{13},E)\notin \tau$, $(F_{2},E)\tilde{\cup }(F_{13},E)\notin \tau$, $(F_{2},E)\tilde{\cap }(F_{14},E)\notin \tau$, and many of soft sets belong to the family  *τ*  and their soft intersection and soft union do not exist in *τ*. Consequently, [1, Examples 27, 28, 29, 30] also are incorrect. Example  3.3 in [2] already proved the results in [1, Remark 12] and [2, Examples 4.3 and 4.4] also proved the results in [1, Remark 26].
